# CUL4A-DDB1-circRFWD2 E3 ligase complex mediates the ubiquitination of p27 to promote multiple myeloma proliferation

**DOI:** 10.1186/s40164-024-00582-8

**Published:** 2024-11-22

**Authors:** Jie Min, Jialei Mao, Hui Shi, Yumeng Peng, Xiaoning Xu, Mengjie Guo, Xiaozhu Tang, Ye Yang, Chunyan Gu

**Affiliations:** 1 Kunshan Hospital of Chinese Medicine, Affiliated Hospital of Nanjing University of Chinese Medicine, Kunshan, China; 2 School of Medicine, Nanjing University of Chinese Medicine, Nanjing, China

**Keywords:** Multiple myeloma, circRFWD2, E3 ligase, p27, Ubiquitination

## Abstract

Multiple myeloma (MM) is an incurable disease characterized by the abnormal expansion of plasma cells in the bone marrow (BM). Numerous studies have shown that BM tumor cells can influence the tumor microenvironment (TME) through communication with extracellular vesicle circular RNAs (circRNAs), a type of noncoding RNA. Our study revealed that a circular RNA, circRFWD2 (hsa_circ_0015361), is expressed by MM cells and translated into a new protein, circRFWD2_369aa. We found that elevated levels of circRFWD2_369aa in MM peripheral blood samples were closely associated with poor outcomes in MM patients. Further investigation revealed that circRFWD2 promoted the degradation of p27 through the ubiquitination pathway, leading to increased proliferation of MM cells. We also confirmed the interaction between circRFWD2 and its downstream genes DDB1 and CUL4A, indicating that circRFWD2 could form an E3 ligase complex with other genes to mediate the ubiquitination of p27. Notably, the protein translated by a circular RNA of RFWD2 can also function as an E3 ligase. Our study highlights the potential of circRFWD2 as a biomarker for MM, which may improve the sensitivity and specificity of diagnosis and efficacy analyses.

**Supplementary Information:**

The online version contains supplementary material available at 10.1186/s40164-024-00582-8.

To the Editor,

Multiple myeloma (MM) is a hematological malignancy that heavily relies on the bone marrow (BM) microenvironment. The interaction between MM cells and other cells in the BM is crucial for the development of MM treatments [1]. Recent research has shown that MM cells can influence the tumor microenvironment by exchanging information through circular RNA, miRNA, lncRNA, and exosomes [2–4]. Previous studies have also demonstrated the potential of microvesicles in monitoring MM tumor load [5]. CircRNAs are currently being investigated as potential biomarkers, and their roles in tumors, including biogenesis, epigenetic regulation, and degradation, have become a new hotspot in the field of noncoding RNA research [6]. Several studies have suggested that circRNAs may even have the ability to be translated into previously unknown protein isoforms [7–9]. The gene Ring Finger and WD Repeat Domain 2 (RFWD2, also known as COP1) is located at chromosomal position 1q25 and is a critical regulator of p27 (Kip1), which affects the proliferation and drug resistance of MM cells by mediating the ubiquitination degradation of p27 [10]. In this study, we aimed to explore the functions and regulatory mechanism of a circular RNA generated from RFWD2, called circRFWD2 (hsa_circ_0015361), in MM.

## Increased expression of circRFWD2 is associated with poor survival and aggressive proliferation in MM

**Fig. 1:**
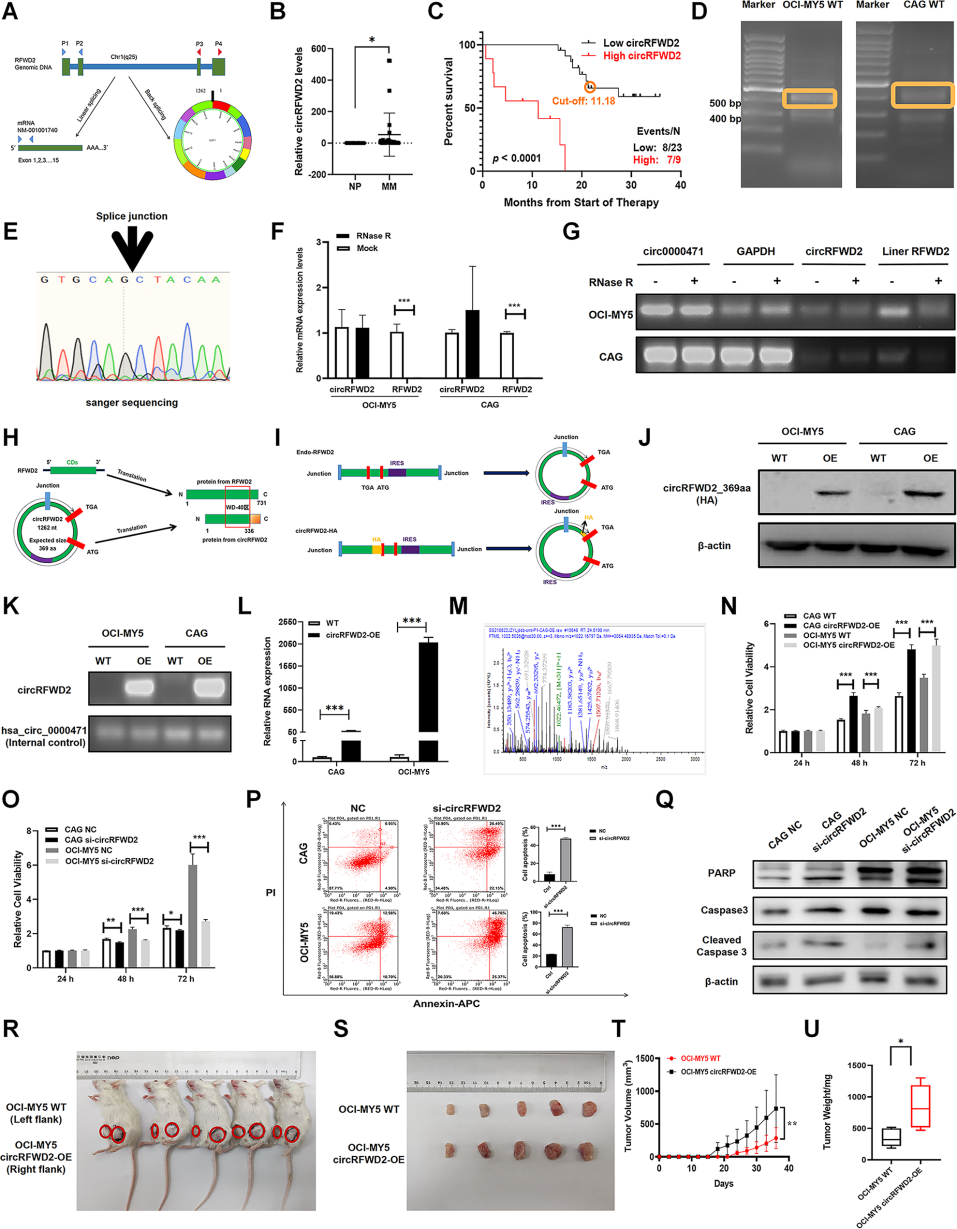
Increased expression of circRFWD2 is associated with poor survival and aggressive proliferation in MM. (**A**) Illustration of the annotated genomic region of RFWD2, the putative different RNA splicing forms, and the validation strategy for circular exons 3 to 15 (circRFWD2). (**B**) The mRNA levels of circRFWD2 were significantly higher in the NDMM group compared to the NP group (*p* < 0.05). (**C**) MM patients with increased expression of circRFWD2 exhibited significantly inferior EFS (*p* < 0.0001). (**D**) Back splicing of exons 3 and 15 of the RFWD2 gene resulted in the formation of an endogenous circRNA in CAG and OCI-MY5 WT cells. (**E**) Sanger sequencing following PCR was used to confirm the “head-to-tail” splicing of circRFWD2 in MM cells via the indicated divergent flanking primers. (**F-G**) RT-PCR and qRT-PCR were used to determine the mRNA levels of circRFWD2 and linear RFWD2 ± RNase R. (**H**) The analysis of ORF revealed that less than a complete circle of circRFWD2 is translated into the novel 369-aa protein. (**I**) To construct the pCL5-HA-circRFWD2 plasmid, the HA tag was added before the TGA (termination codon) according to the translation method of circRFWD2. (**J**) WB analysis showed the overexpression of circRFWD2_369aa in OCI-MY5 and CAG cells, as detected by an HA-tag antibody. (**K-L**) RT-PCR and qRT-PCR were used to confirm the overexpression of circRFWD2. (**M**) Specific peptide fragments identified from circRFWD2_369aa. (**N-O**) A CCK-8 assay was used to examine the proliferation rate of OCI-MY5 and CAG cells in which circRFWD2 was overexpressed or knocked down. (**P**) Annexin APC/PI staining combined with flow cytometry detected the effect of decreased circRFWD2 expression on apoptosis in MM cells. (**Q**) The expression levels of PARP and cleaved Caspase3 were increased in si-circRFWD2 cells. (**R-S**) Photographic images of xenograft mice at day 36 and xenografts from SCID/NOD mice. (**T**) Time course of tumor growth in SCID/NOD mice. (**U**) Tumor weights of the mice in WT and circRFWD2-OE groups at day 36 after injection of MM cells. The data are presented as mean ± SD. *p* < 0.05(*), *p* < 0.01(**), and *p* < 0.001(***) indicate statistically significant differences. NC: normal control

To confirm the presence of endogenous circRFWD2 (hsa_circ_0015361), which is formed by exons 3 and 15 of the RFWD2 gene, we designed divergent primers to detect the back-spliced forms of RFWD2 (Fig. 1A). We collected serum samples from 32 patients with newly diagnosed multiple myeloma (NDMM) and 30 healthy individuals (NP). Interestingly, circRFWD2 was significantly more abundant in MM patients than healthy individuals (*p* < 0.05). Furthermore, we observed that MM patients with higher levels of circRFWD2 presented significantly inferior event-free survival (EFS) (*p* < 0.0001) (Fig. 1B, C; Additional file 2: Table S1). We also confirmed the presence of endogenous circRFWD2 in wild-type (WT) CAG and OCI-MY5 cells (Fig. 1D). By using Sanger sequencing, we identified the circRFWD2 junction site, further confirming the presence of circRFWD2 (Fig. 1E). To investigate the stability of circRFWD2, we designed convergent primers to detect the linear form of RFWD2 mRNA. After RNase R digestion, the linear form was significantly degraded (*p* < 0.001), whereas circRFWD2 remained stable, providing strong evidence for its existence and stability (Fig. 1F, G).

The analysis of the open reading frame (ORF) indicated that it takes less than a complete rotation of circRFWD2 to translate the novel 369-aa protein, referred to as circRFWD2_369aa in this study (Fig. 1H). To validate the prediction results, we overexpressed circRFWD2_369aa by inserting its sequence into a plasmid with an HA tag located before the termination codon (Fig. 1I). Lentivirus-transfected MM cell lines stably overexpressing circRFWD2 coding cDNA were constructed in both CAG and OCI-MY5 cells. The western blot (WB) method successfully detected circRFWD2_369aa at the expected size in CAG and OCI-MY5 cells (Fig. 1J). As expected, qPCR revealed increased levels of circRFWD2 in circRFWD2-overexpression (circRFWD2-OE) cells (Fig. 1K, L). In addition, mass spectrometry (MS) analysis identified the peptide fragments from circRFWD2_369aa, further confirming the coding ability of endogenous circRFWD2 (Fig. 1M). Cell Counting Kit-8 (CCK-8) experiments demonstrated that increased circRFWD2 expression significantly increased cell proliferation in CAG and OCI-MY5 cells (*p* < 0.001), whereas silencing circRFWD2 with a specific small interfering RNA (siRNA) significantly inhibited MM cell proliferation (*p* < 0.001) (Fig. 1N, O). Flow cytometry experiments confirmed that inducible downregulation of circRFWD2 induced cell apoptosis (*p* < 0.001) (Fig. 1P). Furthermore, elevated levels of PARP and cleaved Caspase3 were detected in si-circRFWD2 cells (Fig. 1Q).

To further investigate these findings in vivo, we subcutaneously injected OCI-MY5 WT and OCI-MY5 circRFWD2-OE cells into SCID/NOD mice. After 5 weeks, the tumors formed by OCI-MY5 WT cells were significantly smaller than those formed by OCI-MY5 circRFWD2-OE cells (Fig. 1R, S). The tumor growth curve clearly showed that the average volume of OCI-MY5 WT tumors significantly lagged behind that of circRFWD2-OE tumors (*p* < 0.01) (Fig. 1T). The tumor weight analysis supported the tumor volume data (*p* < 0.05) (Fig. 1U). These results suggest that elevated levels of circRFWD2 are associated with poor survival in MM patients and promote MM cell proliferation both in vitro and in vivo.

## circRFWD2 mediates ubiquitination degradation of p27 by forming an E3 ligase protein complex

**Fig. 2:**
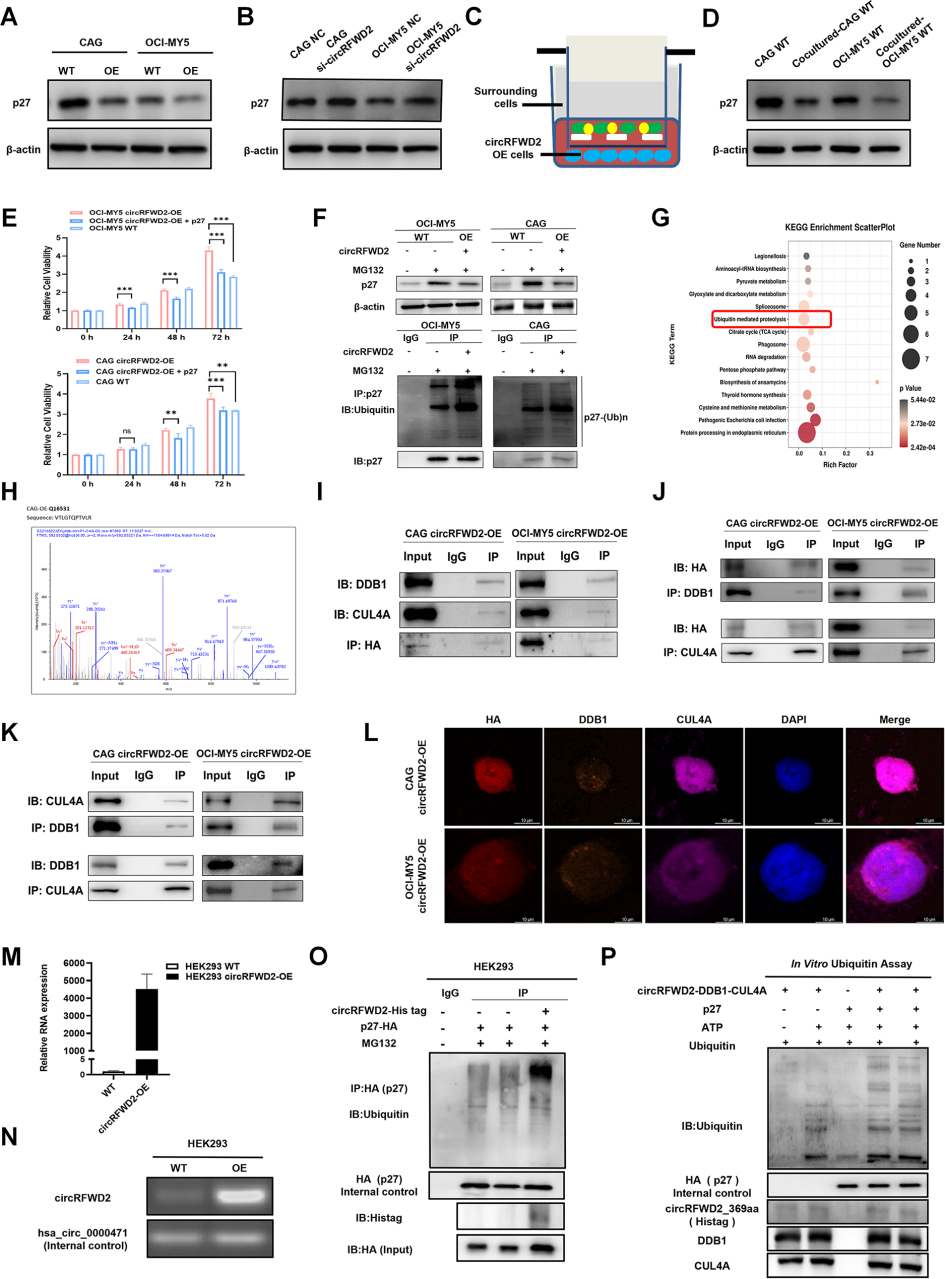
CircRFWD2 mediates ubiquitination degradation of p27 by forming an E3 ligase protein complex. (**A-B**) Overexpression of circRFWD2 led to decreased p27 expression, whereas knockdown of circRFWD2 resulted in increased p27 expression. (**C**) Schematic diagram of the coculture experiment. (**D**) The expression level of p27 was decreased in cocultured WT MM cells. (**E**) CCK-8 experiment showed that the viability and growth of circRFWD2-OE cells were suppressed when cocultured with p27-OE HEK293 cells, compared to non-cocultured cells. CAG and OCI WT cells were used as controls for cell viability and proliferation compared to circRFWD2-OE cells. (**F**) CircRFWD2 affected the protein expression level of p27 through the ubiquitination pathway. OCI-MY5 and CAG cells with or without circRFWD2 overexpression were collected at 12 h after treatment with MG132, a proteasome inhibitor. A ubiquitination assay was performed using anti-p27 magnetic beads, and the ubiquitination levels of p27 were measured using a ubiquitin antibody. (**G**) Schematic diagram of KEGG enrichment analysis. (**H**) The peptide fragments derived from the DDB1 gene (Q16531) are closely related to ubiquitination and are significantly abundant in MM cells. (**I-K**) Co-IP experiments confirmed the interaction between circRFWD2, DDB1, and CUL4A. (**L**) Three key components were labeled with different immunofluorescent dyes in circRFWD2-OE MM cells simultaneously. The overlapping localization of fluorescence signals within cells also indicated their interaction. IF staining revealed that circRFWD2-HA co-localized with DDB1 and CUL4A in MM cells. Scale bar: 100 μm. (**M-N**) The pLC5-circRFWD2-His tag plasmid was successfully constructed. (**O**) Synchronous transient transfection of circRFWD2-His tag and p27-HA plasmids was performed in HEK293 cells, and single transfection of p27-HA and synchronous transient transfection of pLC5-ciR (empty vector) with p27-HA plasmids were used as control groups. Upon transfection of the corresponding plasmids, the ubiquitination and degradation of p27 were followed by treatment with MG132 for 12 h. WB results showed that circRFWD2 overexpression increased the level of ubiquitinated exogenous p27. (**P**) The in vitro ubiquitination experiments revealed that the CUL4A-DDB1-circRFWD2 complex can undergo autoubiquitination and mediate the autoubiquitination of p27

Based on our understanding that RFWD2 can mediate the degradation of p27 through ubiquitination, we conducted experiments to investigate the effects of overexpressing and knocking down circRFWD2 on the expression level of p27 protein in MM cells. Our study revealed that circRFWD2 overexpression led to a decrease in p27 protein levels, whereas circRFWD2 knockdown increased p27 expression in WT MM cells (Fig. 2A, B). To further examine the impact of circRFWD2 on regulating p27 expression, we cocultured CAG and OCI-MY5 WT cells with corresponding circRFWD2-OE cells via a transwell system. We found a significant decrease in p27 expression in the cocultured WT MM cells compared to the non-cocultured cells (Fig. 2C, D). Based on the findings presented above, we attempted to rescue the expression of p27 in circRFWD2-OE MM cells and determine if increasing p27 levels could counteract the increased cell viability and growth caused by circRFWD2 overexpression. Therefore, we conducted a coculture experiment in which circRFWD2-OE CAG and OCI-MY5 cells were cocultured with p27-OE HEK293 cells for 48 h. We then measured the viability of these cells. The results of the CCK-8 assay indicated that increased expression of p27 in the tumor microenvironment suppressed the viability and growth of circRFWD2-OE cells compared to those non-cocultured cells (Fig. 2E). Furthermore, we delved deeper into the pathway through which circRFWD2 regulates the expression level of p27. Our experiments showed that HA-tagged circRFWD2 increased the level of ubiquitination and led to the degradation of p27, indicating that circRFWD2 regulates p27 expression by promoting its ubiquitination and degradation through the ubiquitination pathway (Fig. 2F).

To better understand how circRFWD2 regulates p27 and its ubiquitination levels in cells, we conducted Co-IP/MS experiments in CAG WT and CAG circRFWD2-HA-OE cells via HA antibodies. KEGG analysis from mass spectrometry revealed that the ubiquitination pathway was enriched, and that DDB1 was enriched by the anti-HA antibody in CAG-OE cells (Fig. 2G, H). The peptide fragment of DDB1 identified via MS (PXD number: PXD030089) is shown in Fig. 2H. DDB1 is a crucial structural protein in the Cullin4 (CUL4) ubiquitin ligase complex, responsible for recognizing and ubiquitinating substrate proteins through proteins containing the WD-40 domain. CRL4^CUL4A/DDB1^ is a well-defined E3 ubiquitin ligase [11]. Co-IP experiments confirmed the interaction between circRFWD2_369aa, DDB1, and CUL4A (Fig. 2I, J, K). Additionally, immunofluorescence (IF) staining for circRFWD2_369aa-HA, DDB1, CUL4A, and DAPI revealed the colocalization of these proteins in MM cells (Fig. 2L), indicating that circRFWD2_369aa not only regulates the expression and ubiquitination levels of p27, but also may form complexes with DDB1 and CUL4A.

To further investigate the regulatory role of circRFWD2_369aa on p27, we constructed and validated a circRFWD2-His tag-OE plasmid (Fig. 2M, N). Then, we performed a synchronous transient transfection of circRFWD2-His tag and p27-HA plasmids in HEK293 cells, with single transfection of p27-HA and synchronous transient transfection of pLC5-ciR (empty vector) with p27-HA plasmids as controls. WB results demonstrated that overexpression of circRFWD2 increased the level of ubiquitinated exogenous p27 (Fig. 2O). Furthermore, the in vitro ubiquitination experiments demonstrated the ability of the complex formed by circRFWD2_369aa, DDB1, and CUL4A to undergo autoubiquitination and mediate the ubiquitination of p27 (Fig. 2P; Additional file 2: Table S4).

In summary, our research provides a new perspective on the role of circRNA in tumor occurrence and development. We demonstrate the potential of circRFWD2 (hsa_circ_0015361) as a biomarker for poor prognosis in MM patients. Our findings reveal that circRFWD2_369aa plays a crucial role in regulating the expression and ubiquitination of p27 in MM cells by forming complexes with DDB1 and CUL4A as an E3 ligase.

## Electronic supplementary material

Below is the link to the electronic supplementary material.

Supplementary Material 1

Supplementary Material 2

## Data Availability

The datasets analyzed in the current study are available upon reasonable request from the corresponding author. The MS proteomics data have been deposited to the ProteomeXchange Consortium via the iProX partner repository with the dataset identifier PXD030089. The raw data of MS can be found here: https://www.iprox.cn/page/project.html? id=IPX0003745000.

## Abbreviations

MM: Multiple myeloma
BM: Bone marrow
TME: Tumor microenvironment
circRNAs: circular RNAs
RFWD2: Ring Finger and WD Repeat Domain 2
DDB1: And CUL4 associated factor
CRL4: Cullin-Ring ligase 4
SCF: SKP1-CUL1-F-Box
ORF: Open reading frame
IRES: Internal ribosome entry site
NDMM: Newly Diagnosed Multiple Myeloma
NP: Normal individuals
EFS: Event free survival
CCK-8: Cell Counting Kit-8;
siRNA: Small interfering RNA;
MS: Mass spectrometry
IF: Immunofluorescence;
CUL4: Cullin 4;
WDRs: WD40 repeat proteins
