# Supplementary material for: CUL4A-DDB1-circRFWD2 E3 ligase complex mediates the ubiquitination of p27 to promote multiple myeloma proliferation
Source: Exp Hematol Oncol. 2024 Nov 22;13:116. doi: 10.1186/s40164-024-00582-8 (PMC11583565; doi:10.1186/s40164-024-00582-8)
Supplement: Supplementary file 1 — Supplementary Material 1 [file 40164_2024_582_MOESM1_ESM.docx]

**Supplementary Materials and Methods**

***Gene expression profiling***

The gene expression profiling (GEP) of MM patients was obtained from the GEO database as previously described [1,2]. The total therapy 2 (TT2, GSE2658) and the assessment of proteasome inhibition for extending remission (APEX, GSE9782) patient cohorts were included in analyses **(Additional file 1: Fig. S2A, B)**.

***Antibodies and reagents***

The primary antibodies used in this study were diluted at a ratio of 1:1000 and included HA (AE008, ABclonal, China), poly ADPribose polymerase (PARP) (9542S, Cell Signaling Technology, USA), β-actin (4970S, Cell Signaling Technology, USA), rabbit IgG (a7016) and mouse IgG (a7028; Beyotime Institute of Biotechnology, China), Ubiquitin (10201-2-AP, ProteinTech Group, China), Cullin 4A(A2882, ABclonal, China), DDB1(ET1706-2, HUABIO, China), RFWD2 (A10463, ABclonal, China). Propidiumiodide (PI) was purchased from XunBei (Nanjing, China), and Annexin-V-FITC was purchased from Biolegend (San Diego, CA, USA).

***Cell lines and cell culture***

Human MM cell lines OCI-MY5 and CAG were cultured in RPMI-1640 (Biological Industries, Israel). HEK293 cells were cultured in DMEM (Thermo Fisher Scientific, USA). Culture medium was added with fetal bovine serum (10%, Gibco, USA), penicillin (100 U/mL, HyClone, USA), and streptomycin (100 μg/mL, HyClone, USA), which was changed every 2-3 days. All cells were cultured in 100 mm dishes at 37°C in a 5% CO_2_ incubator.

***Plasmids and transfection***

A commercially available circular RNA expression vector pLC5-ciR (GS0104, Guangzhou Geneseed Biotech Co, China) was used to generate a circRFWD2-overexpression vector. To induce circularization in vivo, side flanking repeat sequences and SA/SD sequences were added to both sides of the 1262 nt sequences (OV-circRFWD2). The front circular frame contained the endogenous flanking genomic sequences with the EcoRI restriction enzyme site, and the back-circular frame included part of the inverted upstream sequence with the BamHI site.

Lentiviruses were produced by co-transfection of the expression vector of interest with the packaging plasmids psPAX2 and pMD2G (Addgene) into HEK293 cells using Hief Trans™ Liposomal Transfection Reagent (Cat#40802, YEASEN, China). The virus supernatant was collected after 48 h. Transfected MM cells were screened by puromycin. The qPCR and WB methods were performed to examine overexpression efficiency.

***Cell proliferation and apoptosis assays***

Cell Counting Kit-8 (CCK-8) (CCK-810-02, SinoMol, China) was used to measure cell proliferation and cell viability at 24, 48, and 72 h in 96-well plates. At the corresponding time points, 10 μL of CCK8 solution was added to each well, and the plates were incubated for an additional 4 h before testing **(Fig. 1N, O; Fig. 2E)**. The cells were collected 48 h after the transfection with si-circRFWD2. A total of 1×10^6^ cells were washed with PBS twice and resuspended using a binding buffer. PI and Annexin-V-FITC were added, and the cells were incubated for 15 min before being analyzed using flow cytometry equipped with the Guava easyCyte System (Merck Millipore, Darmstadt, Germany). The total cell apoptosis ratio was calculated by adding the early and late apoptosis ratios. The experiments were repeated three times **(Fig. 1P)**.

***RNase R treatment***

RNase R (Epicentre Biotechnologies, Madison, WI) was used to degrade linear mRNA. In brief, RNAs were extracted from OCI-MY5 and CAG cells, and divided into two parts, one for RNase R digestion and another for control with digestion buffer only. RNase R treatment (20 U/μL) was performed on total RNA (20 μg) at 37°C for 15 min.

***RT- qPCR***

Total RNA was isolated from MM cells using TRIeasy^TM^ Total RNA Extraction Reagent (YEASEN, Shanghai). The Hifair1st Strand cDNA Synthesis SuperMix was utilized to reverse transcribe 500ng of purified RNA. PCR samples were prepared with diluted cDNA (1:30), 5μL SYBR Green PCR master mix (YEASEN, Shanghai), and 0.2 μM each of the forward and reverse primers in a total volume of 10 μL. Quantitative PCR (qPCR) reaction procedure was conducted as follows: pre-denaturation temperature, 95°C, 3 min; denaturation temperature, 95°C, 10 s; annealing temperature, 60°C, 59 s; a total of 40cycles. The qPCR was performed in an Analytikjena qPCR soft 4.0 (Germany). The relative expression level of target genes was calculated using the 2−∆∆CT method and graphed as fold change (2−∆∆CT) from the control. The information of primer sequences is listed in **Additional file 2:** **Table S3**.

***Co-immunoprecipitation (Co-IP)***

Pierce Direct Magnetic IP/CO Immunoprecipitation (Co-IP) Kit (Thermo Scientific) was used to perform the Co-IP assay according to the manufacturer’s instructions.

***Recombinant plasmid construction***

PCR was conducted using the Phanta Super-Fidelity DNA Polymerase kit (Vazyme, Nanjing). Gel extraction was carried out using the Agarose Gel Recovery Kit from Tiangen Biochemical Co. The overlapping PCR reactions were also performed using the Phanta Super-Fidelity DNA Polymerase kit from Nanjing Novozymes. The product of the touch-down PCR reaction was then recombined with the circular RNA expression vector pLC5-ciR by using the CloneUFO® One Step Cloning Kit (ATG, Nanjing).

***MM- xenografted SCID/NOD mouse model***

Wild type (WT) and circRFWD2-overexpression (circRFWD2-OE) MM cells were injected subcutaneously into the left and right abdominal flanks of 6~8-week-old SCID/NOD mice at a density of 1×10^6^ cells per injection. Tumor diameter was measured using the calipers. The mice were sacrificed when the tumor diameter reached 20 mm. The tumor tissues were collected, weighed, and photographed. All animal studies were conducted by the Government-published recommendations for the Care and Use of Laboratory Animals and approved by the Institutional Ethics Review Boards of Nanjing University of Chinese Medicine (Ethics Registration no. 201905A003).

***In vitro ubiquitination experiment***

E3 Ligase Auto-Ubiquitylation Assay (ab139469, Abcam) was utilized to measure the ubiquitination level of p27 *in vitro*. The assay reagents were thoroughly combined and mixed, then incubated at 37°C for 1 h. After incubation, the reaction was quenched with 2×SDS-PAGE gel loading buffer and analyzed by WB. Specific volumes of each reagent and the reaction system can be found in **Additional file 2: Table S4**.

***Statistical analysis***

Statistical analyses were conducted using GraphPad Prism 6.01 software, and all values were reported as mean ± SD unless otherwise specified. A two-tailed Student’s t-test was used for two-group comparisons, while a one-way analysis of variance was used for multiple comparisons to determine the significance between experimental groups. The Kaplan-Meier method and Log-rank test were utilized to determine the survival rate of patients with MM. Statistical significance was denoted by *p* < 0.05 (*), *p* < 0.01 (**) and *p* < 0.001 (***).

**Supplementary Results**

**Overexpression and knockdown of circRFWD2 play a crucial role in determining its impact on MM growth**

We not only discovered a significant correlation between the expression of circRFWD2 and poor prognosis in 32 samples from patients with newly diagnosed multiple myeloma (NDMM) (Fig. 1C), but also delved deeper into the relationship between the level of circRFWD2 and the various cytogenetic abnormalities present in MM. Interestingly, we found that the expression level of circRFWD2 was associated with 1q21 gain/amp, a typical cytogenetic aberration. The experimental results showed that there was no significant difference in the expression of circRFWD2 between the 1q21 negative group and the positive group, while the expression in the 1q21 amp group was significantly higher than that in the gain group (Additional file 1: Fig. S1A, B). Based on the circbase database, our initial prediction indicated the presence of a secreted circular RNA fragment of the RFWD2 gene, circRFWD2, with a length of 1262 bp and containing 13 exons and a putative internal ribosome entry site (IRES) sequence. To confirm the existence of this endogenous circRNA, we designed divergent primers specifically targeting the circular form of RFWD2 mRNA. The resulting PCR amplicon, 412 bp in length, contains the "head-to-tail" splicing sites of circRFWD2, which we have indicated the predicted product by circling the band on the gel. The primer sequences were verified using Check Primer to ensure high specificity in distinguishing it from other circRNAs (Fig. 1D; Additional file 1: Fig. S1C). We successfully amplified the PCR products using the divergent primer in various MM cell lines (Additional file 1: Fig. S1D). The knockdown and overexpression efficiency of circRFWD2 were evaluated in OCI-MY5 and CAG cell lines, as well as the baseline levels of circRFWD2 in these cell lines were determined (Additional file 1: Fig. S1E, F, G). The knockdown efficiency of circRFWD2 is shown in Fig. S1E using RT-qPCR (*p* <0.01). The corresponding lanes in Fig. S1F show the RT-qPCR product, confirming the baseline level of circRFWD2. The internal control gene for circRNAs was hsa_circ_0000471. We also used the divergent primers to detect the same batch of samples in Fig. 1K, and the bands of the overexpression amplicons had a higher fluorescence intensity (Additional file 1: Fig. S1G).

In addition, we detected the expression of RFWD2 to ensure the specificity of overexpression of only the circular form of the protein. As shown in Fig. S1H, the overexpression of circRFWD2 did not increase RFWD2 expression, confirming the specificity of the overexpression of the circular protein. To assess the effects of circRFWD2 overexpression, we utilized a clonogenic soft agar assay in both circRFWD2-OE and WT cells (CAG & OCI-MY5) for a long-term observation. Our results showed a significant increase in clonal formation in circRFWD2-OE cells compared to WT cells at the same time point (Additional file 1: Fig. S1I). The additional photographic images of xenograft mice were also included in Fig. S1J to provide further evidence for the subcutaneous tumor formation experiment. Furthermore, the expression of circRFWD2 was confirmed using an HA-tag antibody in the isolated tumors (Additional file 1: Fig. S1K).

**The interaction between circRFWD2 and p27 regulates the development of MM, while the CUL4A-DDB1-circRFWD2 complex plays a crucial role in the ubiquitination pathway**

The probe 207805_at, designed based on p27, was used to analyze whether p27 is also a significant prognostic factor in MM. The decreased expression of p27 was linked to poor survival rates in patients with MM. In both the TT2 and APEX patient cohorts, a decrease in p27 mRNA expression was found to be associated with lower overall survival (OS) (Additional file 1: Fig. S2A, B). We collected serum samples from 31 patients with RRMM and 38 healthy individuals (NP). CircRFWD2 was significantly more abundant in MM patients compared to normal individuals (*p* < 0.001). Furthermore, MM patients with higher levels of circRFWD2 exhibited a significantly inferior Event Free Survival (EFS) (*p* < 0.0001) (Additional file 1: Fig. S2C, D; Additional file 2: Table S2). We further validated the relative expression levels of p27 in the 31 samples. Among these samples, 3 in the lower expression group did not show any detectable levels of p27, while 6 samples in the higher expression group also did not show any detectable levels. Our findings demonstrated a significant increase in p27 expression in the low circRFWD2 expression group (*p* < 0.001) (Additional file 1: Fig. S2E). Additionally, we observed a negative correlation between the expression of circRFWD2 and p27 in MM patients (Additional file 1: Fig. S2F). This suggests an inverse relationship between circRFWD2 and p27 expression in patients with MM.

This is particularly relevant because the CRL4-E3 ligase complex plays a crucial role in mediating the effects of IMiDs, which are currently the primary treatment for MM. To rule out the possibility of gene redundancy between circRFWD2 and CRBN, we examined the interaction between the components of the CUL4A-DDB1-circRFWD2 complex. The results of WB and Co-IP experiments showed no visible interaction between them (Additional file 1: Fig. S2G), providing evidence for the function of circRFWD2 in the CUL4A-DDB1-circRFWD2 core complex.

**Supplementary Discussion**

The bone marrow (BM) microenvironment is a complex and dynamic ecological niche that provides an optimal environment for the growth of myeloma, making it an important site for the progression of myeloma [3]. Recent research has shown that bone marrow tumor cells can influence the tumor microenvironment through circRNAs [4]. The widespread expression of circRNAs in tissues and their specificity at different developmental stages make them more effective as biomarkers compared to other molecules in terms of analysis specificity and accuracy. Many circRNAs have already been successfully used as biomarkers for various human diseases. Liquid biopsy, a revolutionary tool in disease detection, has provided significant support for the diagnosis, prognosis, and treatment of human diseases. Therefore, it is essential to establish the analytical effectiveness and clinical utility of circRNAs. In this study, we discovered that high expression of circRFWD2 is closely associated with poor prognosis in MM patients. Considering its circRNA characteristics, biological functions, and advancements in drug delivery methods and gene therapy, there is potential to utilize si-circRFWD2 as a therapeutic RNA loaded into natural delivery tools such as exosomes. This may be a promising treatment strategy for MM, paving the way for new possibilities in clinical treatment.

After conducting research on the promoting effect of circRFWD2_369aa on MM cell proliferation, further investigation was carried out to determine its specific mechanism of action. As RFWD2 is an E3 ubiquitin ligase that mediates the ubiquitination of p27 [5], and circRFWD2 shares partial similarities with its functional domain, additional validation was performed to confirm whether circRFWD2_369aa also plays a similar role in regulating p27 expression. As a classic tumor suppressor gene, the downregulation of the p27 protein expression level in MM is closely associated with poor prognosis for MM patients [6, 7]. Further experiments on ubiquitination-related experiments revealed that circRFWD2 can affect p27 expression levels through the ubiquitin pathway. Moreover, a precise interaction between circRFWD2 and DDB1, CUL4A was discovered, suggesting that circRFWD2 may not only regulate the expression and ubiquitination levels of p27, but may also form complexes with DDB1 and CUL4A, similar to the function of the CUL3 E3 ligase complex and SCF ubiquitin ligase complex in the ubiquitin E3 ligase family, indicating that circRFWD2 plays a crucial role in the ubiquitin proteasome pathway.

RFWD2 is an E3 ubiquitin ligase that plays a role in various biological processes, including promoting protein ubiquitination and acting as a DDB1 and CUL4 associated factor (DCAF) protein in the formation of a CRL4-DCAF ubiquitin E3 ligase complex to target substrates for ubiquitination [8]. Research has shown that DDB1 is a crucial structural component of the CUL4 ubiquitin ligase complex, responsible for recognizing and ubiquitinating substrate proteins through proteins containing the WD-40 domain. Additionally, it has been suggested that the CUL4-DDB1 ligase utilizes WD40 repeat proteins (WDRs) as molecular adapters for substrate recognition and regulates various biological functions through ubiquitin-dependent protein degradation. In this process, WDR proteins act as crucial substrate-specific adapters [9, 10]. From a structural perspective, circRFWD2 contains three complete WD-40 functional domains, indicating its potential role in recruiting substrates into the CUL4A-DDB1-circRFWD2 core complex. In other words, circRFWD2 may function as a DCAF protein or a WDR protein to form a CRL4 E3 ligase complex and ubiquitinate substrates such as p27 [11].

There is substantial evidence to suggest that the abnormal expression of E3 ubiquitin ligases is closely related to the occurrence of cancer. Overexpression of E3 ubiquitin ligases in cancer patients can increase the risk of chemotherapy resistance and lead to a poor clinical prognosis. In addition to functioning independently, E3 ligases can also form complexes to mediate the ubiquitination degradation of various proteins by forming complexes, including p27, p21, and p57. Researchers have extensively studied p27, which plays a crucial role in binding to CDK, inhibiting its catalytic function, and promoting cell cycle arrest [12]. A decrease in the expression level of p27 may contribute to tumor formation. Therefore, a promising research approach has emerged to stabilize p27 in cancer treatment by inhibiting E3 complexes such as CRL1-SKP2 ligases. The ideal inhibitor would specifically interfere with the interaction between E3 ubiquitin ligase and tumor-inhibiting proteins, as E3 ubiquitin ligase has a similar catalytic domain and the ability to simultaneously target both tumor-promoting and tumor-inhibiting proteins [13]. Even without a ring finger domain, circRFWD2 can still play a role in the ubiquitination degradation of substrate proteins. Further investigation into the binding between its WD40 domain and DDB1, exploring the structural basis of their interaction, and developing corresponding competitive inhibitors to stabilize p27 may be a promising avenue for research [14].

In conclusion, our research has provided a new perspective on the role of circRNA in tumor occurrence and development. Specifically, we have identified circRFWD2 (hsa_circ_0015361) as a potential biomarker for poor prognosis in MM patients. Our findings suggest that circRFWD2 can regulate MM cell proliferation through translation pathways. Furthermore, our study reveals that circRFWD2 plays a crucial role in the expression and ubiquitination level of p27 in MM cells by forming complexes with DDB1 and CUL4A as an E3 ligase. The discovery of circRNA with E3 ligase function opens up new possibilities for identifying biomarkers of MM and developing targeted drugs for the clinical treatment of tumors.

**References:**

1. Zhan F, Huang Y, Colla S, Stewart JP, Hanamura I, Gupta S, et al. The molecular classification of multiple myeloma. Blood. 2006; 108(6):2020-8.
2. Broyl A, Hose D, Lokhorst H, de Knegt Y, Peeters J, Jauch A, et al. Gene expression profiling for molecular classification of multiple myeloma in newly diagnosed patients. Blood. 2010; 116(14):2543-53.
3. Bianchi G, Munshi NC. Pathogenesis beyond the cancer clone(s) in multiple myeloma. Blood. 2015; 125(20):3049-58..
4. Gu C, Wang W, Tang X, Xu T, Zhang Y, Guo M, et al. CHEK1 and circCHEK1_246aa evoke chromosomal instability and induce bone lesion formation in multiple myeloma. Mol Cancer. 2021; 20(1):84.
5. Gu C, Lu T, Wang W, Shao M, Wei R, Guo M, et al. RFWD2 induces cellular proliferation and selective proteasome inhibitor resistance by mediating P27 ubiquitination in multiple myeloma. Leukemia. 2021; 35(6):1803-1807.
6. Zhan F, Colla S, Wu X, Chen B, Stewart JP, Kuehl WM, et al. CKS1B, overexpressed in aggressive disease, regulates multiple myeloma growth and survival through SKP2- and p27Kip1-dependent and -independent mechanisms. Blood. 2007; 109(11):4995-5001..
7. Filipits M, Pohl G, Stranzl T, Kaufmann H, Ackermann J, Gisslinger H, et al. Low p27Kip1 expression is an independent adverse prognostic factor in patients with multiple myeloma. Clin Cancer Res. 2003; 9(2):820-6.
8. Luo D, Chen M, Li Q, Wang K, Wang K, Li J, et al. CUL4B-DDB1-COP1-mediated UTX downregulation promotes colorectal cancer progression. Exp Hematol Oncol. 2023; 12(1):77..
9. Angers S, Li T, Yi X, MacCoss MJ, Moon RT, Zheng N. Molecular architecture and assembly of the DDB1-CUL4A ubiquitin ligase machinery. Nature. 2006; 443(7111):590-3.
10. Higa LA, Wu M, Ye T, Kobayashi R, Sun H, Zhang H. CUL4-DDB1 ubiquitin ligase interacts with multiple WD40-repeat proteins and regulates histone methylation. Nat Cell Biol. 2006; 8(11):1277-83..
11. Marine JC. Spotlight on the role of COP1 in tumorigenesis. Nat Rev Cancer. 2012; 12(7):455-64.
12. Currier AW, Kolb EA, Gorlick RG, Roth ME, Gopalakrishnan V, Sampson VB. p27/Kip1 functions as a tumor suppressor and oncoprotein in osteosarcoma. Sci Rep. 2019; 9(1):6161.
13. Sampson C, Wang Q, Otkur W, Zhao H, Lu Y, Liu X, et al. The roles of E3 ubiquitin ligases in cancer progression and targeted therapy. Clin Transl Med. 2023; 13(3):e1204.
14. Schapira M, Tyers M, Torrent M, Arrowsmith CH. WD40 repeat domain proteins: a novel target class? Nat Rev Drug Discov. 2017; 16(11):773-786.

**Supplementary Figures**

**
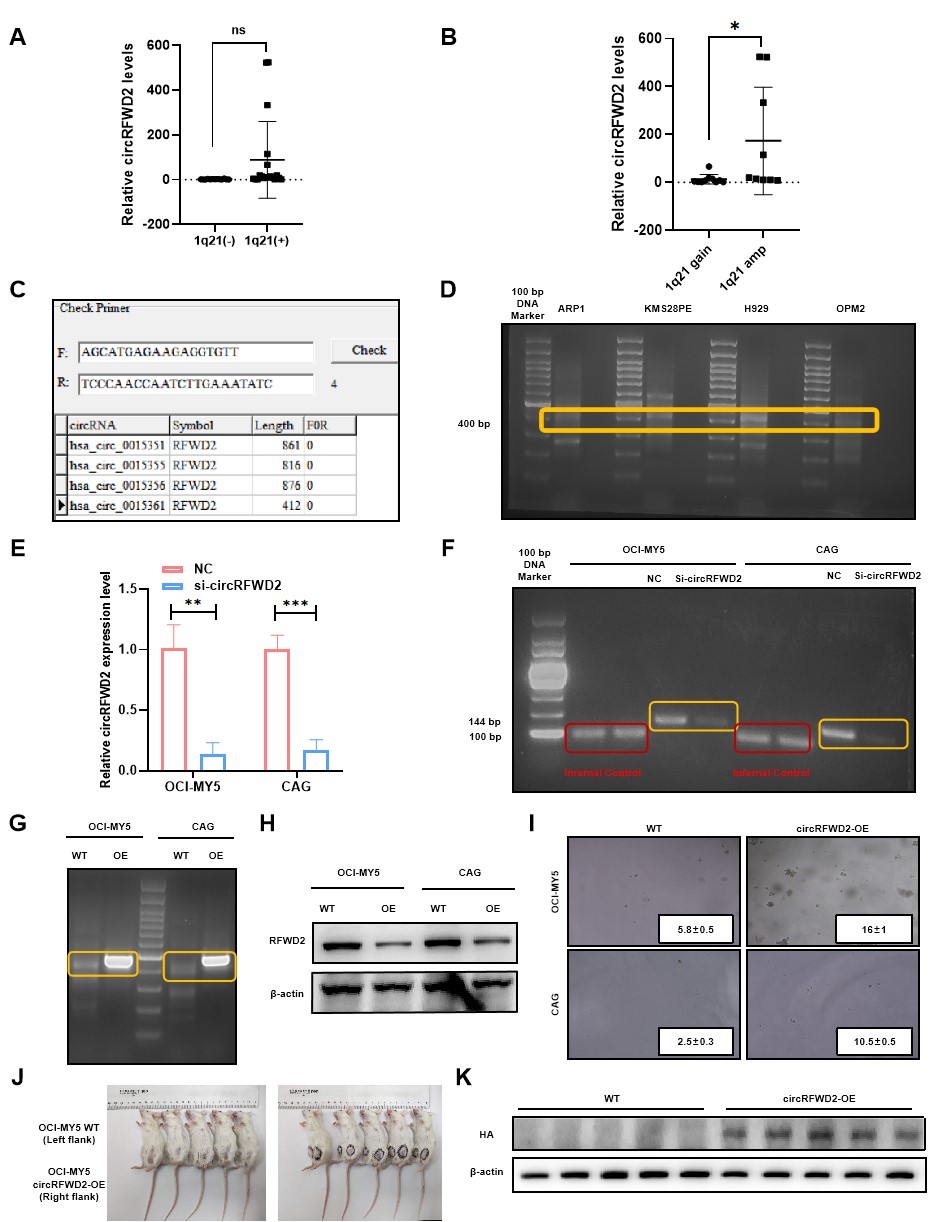
**

**Fig. S1 Overexpression and knockdown of circRFWD2 significantly impact MM growth. (A-B)** The expression level of circRFWD2 was significantly increased in cases with 1q21 gain/amp, a typical cytogenetic aberration. There was no significant difference in the expression of circRFWD2 between the 1q21 negative group and the positive group, but its expression was significantly higher in the 1q21 amp group compared to the gain group. **(C)** The primer designed to detect the circular form of RFWD2 mRNA (hsa_circ_0015361) was verified by Check Primer to have high specificity in distinguishing it from other circRNAs. **(D)** The divergent primer successfully amplified the PCR products in various MM cell lines. **(E-G)** The knockdown and overexpression efficiency of circRFWD2 in OCI-MY5 and CAG cell lines were evaluated. The baseline levels of circRFWD2 in these cell lines were also determined. **(H)** The expression of RFWD2 did not increase with the overexpression of circRFWD2, ensuring the specificity of overexpression of only the circular form of the protein. **(I)** Representative images of soft agar plates showed accelerated clonogenic growth of circRFWD2-OE cells compared to WT cells. **(J)** Additional photographic images of xenograft mice were included to provide further evidence for the subcutaneous tumor formation experiment. **(K)** The expression of circRFWD2 was confirmed using an HA-tag antibody in the isolated tumors. The data are presented as mean ± SD, with statistically significant differences indicated by *p* < 0.05(*), *p* < 0.01(**) and *p* < 0.001(***).

**
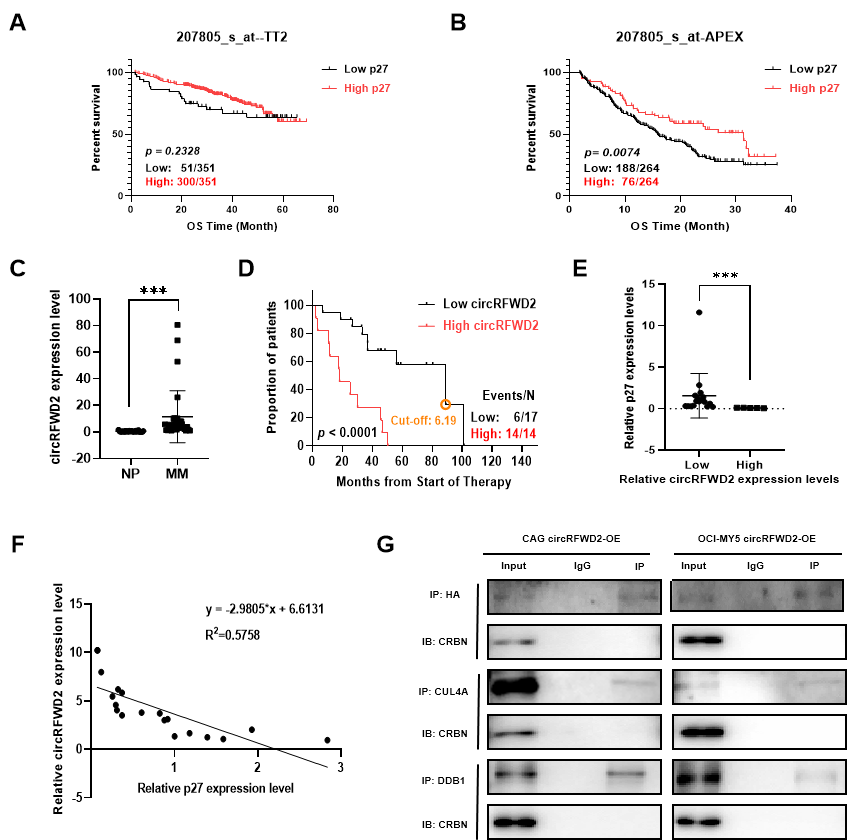
**

**Fig. S2 The interaction of circRFWD2 and p27 controls the development of MM, while the CUL4A-DDB1-circRFWD2 complex plays a crucial role in the ubiquitination pathway. (A-B)** A decrease in p27 mRNA expression was associated with a lower overall survival (OS) in both the TT2 and APEX patient cohorts. **(C-D)** The RRMM group showed significantly higher levels of circRFWD2 mRNA compared to the NP group (*p* < 0.001). MM patients with the higher expression of circRFWD2 exhibited significantly inferior EFS (*p* < 0.0001). **(E)** The group with low circRFWD2 expression showed a significantly higher expression level of p27 (*p* < 0.001). **(F)** circRFWD2 expression was inversely correlated with p27 expression in MM patients. **(G)** The components of the CUL4A-DDB1-circRFWD2 complex did not interact with CRBN. The data are presented as mean ± SD, with statistically significant differences indicated by *p* < 0.05(*), *p* < 0.01(**) and *p* < 0.001(***).
